# Identification and functional analysis of a biflavone as a novel inhibitor of transient receptor potential vanilloid 4-dependent atherogenic processes

**DOI:** 10.1038/s41598-021-87696-9

**Published:** 2021-04-14

**Authors:** Mazen O. Alharbi, Bidisha Dutta, Rishov Goswami, Shweta Sharma, Kai Y. Lei, Shaik O. Rahaman

**Affiliations:** Department of Nutrition and Food Science, University of Maryland, College Park, MD 20742 USA

**Keywords:** Biochemistry, Ion channels, Transient receptor potential channels

## Abstract

Atherosclerosis, a chronic inflammatory disease of large arteries, is the major contributor to the growing burden of cardiovascular disease-related mortality and morbidity. During early atherogenesis, as a result of inflammation and endothelial dysfunction, monocytes transmigrate into the aortic intimal areas, and differentiate into lipid-laden foam cells, a critical process in atherosclerosis. Numerous natural compounds such as flavonoids and polyphenols are known to have anti-inflammatory and anti-atherogenic properties. Herein, using a fluorometric imaging plate reader-supported Ca^2+^ influx assay, we report semi high-throughput screening-based identification of ginkgetin, a biflavone, as a novel inhibitor of transient receptor potential vanilloid 4 (TRPV4)-dependent proatherogenic and inflammatory processes in macrophages. We found that ginkgetin (1) blocks TRPV4-elicited Ca^2+^ influx into macrophages, (2) inhibits oxidized low-density lipoprotein (oxLDL)-induced foam cell formation by suppressing the uptake but not the binding of oxLDL in macrophages, and (3) attenuates oxLDL-induced phosphorylation of JNK2, expression of TRPV4 proteins, and induction of inflammatory mRNAs. Considered all together, the results of this study show that ginkgetin inhibits proatherogenic/inflammatory macrophage function in a TRPV4-dependent manner, thus strengthening the rationale for the use of natural compounds for developing therapeutic and/or chemopreventive molecules.

## Introduction

Atherosclerosis, a progressive inflammatory disease of large arteries, constitutes the foremost contributor to the growing burden of cardiovascular disease-related mortality and morbidity globally^1^. The pivotal initiating event that leads to atherosclerosis involves retention of modified low-density lipoprotein (oxLDL) particles in the subendothelial aortic intimal spaces primarily in arterial branch points^2^. During early atherogenesis, following endothelial dysfunction, circulating blood monocytes transmigrate into the intima areas, and differentiate into tissue macrophages^3^. In aortic intimal spaces, binding and uptake of oxLDL by macrophages aided by scavenger receptors like SRA and CD36 creates a feedforward athero-inflammatory loop resulting in development of lipid-laden foam cells, a critical process in atherosclerosis^2–8^. Cascades of athero-inflammatory events provoked by foam cells lead to the formation of an early fatty streak and eventually the appearance of advanced atherosclerosis lesions characterized by the presence of fibrous cap, calcified tissue, immune cells, matrix proteins, and lipids^2–10^.

Transient Receptor Potential Vanilloid 4 (TRPV4) of the TRPV subfamily, a non-selective, polymodal cation channel permeable to Ca^2+^ is ubiquitously expressed in various cell types including macrophages^11–24^. Previously known as an osmosensor, TRPV4 is now known to respond to multifarious biochemical and biomechanical stimuli including heat, matrix stiffness, osmolarity, growth factors, pH, and 4α phorbol esters^11–24^. TRPV4 is reported to mediate several physiological functions like pain sensation in inflammation and neuropathies^18,22,23^, myofibroblast differentiation and migration ^17,25,26^, and osteoclast differentiation in the bone^27^. TRPV4 mutations have been associated with several channelopathies^28–31^. Recent studies by our group and others have implicated a possible role of this channel in myriad pathological conditions like lung injuries^22,32^, foam cell formation^14,16^, tissue fibrosis^17,33^, foreign body response^13^, and cancer^34,35^. Previously, an atheroprotective role of TRPV4 was shown, in which chemical agonist-induced TRPV4 function in endothelial cells was shown to be associated with activation of eNOS and inhibition of monocyte adhesion to endothelial cells^36^. In contrast, deficiencies in TRPV4 functions have been linked to numerous atheroinflammatory responses including endothelial dysfunction, reduced macrophage foam cell generation, and vascular diseases^14,16,37–39^. Our laboratory has recently identified a proatherogenic role of TRPV4 whereby it regulates oxLDL-induced macrophage foam cell formation. Mechanistically, we showed that TRPV4 affects the uptake but not binding of oxLDL in macrophages in a CD36-independent manner^14^. In another study, we reported a TRPV4-dependent exacerbation of oxLDL-induced foam cell formation in the presence of lipopolysaccharide and increasing matrix stiffness, thus providing a link between mechanosensing and risk of atherosclerosis^16^. Considered all together, these findings suggest TRPV4 as an attractive target in atherosclerosis and other diseases, thus incentivizing the discovery of small molecule inhibitors of TRPV4 that can be translated into clinically effective therapeutics.

The uses of natural compounds in therapeutics have gained prominence, primarily driven by the need for cost effective and biocompatible compounds as compared to the currently existent synthetic drugs. Natural compounds such as flavonoids, alkaloids, polyphenols, and curcuminoids, affect cardiovascular diseases via diverse mechanisms such as inhibition of proinflammatory signals, insulin sensitization, and improving blood lipid profiles by targeting the AMPK, COX1/2, eNOS, and Nrf2 pathways^40–45^. Recent studies by multiple groups have identified two flavonoids, berberine and apigenin, as potential modulators of TRPV4 activity^46,47^. We have developed and used a semi high-throughput screening method to identify potential antagonists of TRPV4 from a library of natural compounds using a Fluorometric Imaging Plate Reader (FLIPR) -based Ca^2+^ influx assay. Herein, we report the identification and functional analysis of ginkgetin, a biflavone, as a novel inhibitor of TRPV4-dependent atherogenic processes in macrophages, thus laying a foundation for further studies of this natural compound as a natural anti-atherosclerotic small molecule compound. Ginkgetin has been reported to display neuroprotective, anti-carcinogenic, and anti-inflammatory roles^48,49^. We report the mechanism of action of ginkgetin against TRPV4 in the setting of macrophage foam cell formation using numerous biochemical and cellular assays.

## Materials and methods

### Animal and cell culture

We purchased congenic C57BL/6 wild type (WT) mice from Charles River Laboratories (Wilmington, Massachusetts, USA). University of Maryland Animal Care and Use Committee guidelines were followed for all animal experiments and the authors complied with ARRIVE guidelines. For isolation of thioglycolate-induced murine resident macrophages (MRM), peritoneal lavage was collected after 4 days of intraperitoneal thioglycolate injection, and cells were maintained in 10% fecal bovine serum (FBS) containing DMEM^7,14,16^. A murine macrophage cell line (RAW264.7), and human dermal fibroblasts (HDF) were purchased from ATCC (Manassas, VA), and were cultured, respectively, with DMEM or MEM (Gibco, Waltham, MA). Murine bone marrow derived macrophages (BMDMs) were harvested from 6- to 7-week old mice, as previously described^14,50,51^. Briefly, femurs from WT and TRPV4 KO mice were collected, and bone marrow of femurs was flushed out with complete DMEM media with 10% FBS and. The suspended bone marrow cells were filtered through a 70 µm strainer (BD bioscience), centrifuged, and maintained in DMEM medium supplemented with M-CSF (25 ng/mL) for 7–8 days at 37 °C to allow differentiation into macrophages.

### Reagents

Ginkgetin and GSK1016790A (GSK101) were purchased from Sigma-Aldrich (St. Louis, MO), and FLIPR calcium 6 assay kit was obtained from Molecular Device (Sunnyvale, CA). Human native LDL (nLDL) was purchased from Stemcell Technologies (Vancouver, Canada). We incubated nLDL with 5 µM CuSO_4_ for 12 h at 37 °C to prepare copper-oxidized LDL (oxLDL)^7,14,16^. Fluorescent dye-labeled, DiI-oxLDL, was purchased from Invitrogen (Carlsbad, CA), and MCSF from R&D (Minneapolis, MN). Antibodies for FACS analysis were: TRPV4 (Millipore; Burlington, MA), CD36 (R&D; Minneapolis, MN), TLR4 and TLR6 (Novus Biologicals; Centennial, CO). PE-conjugated secondary antibodies were from R&D (Minneapolis, MN), and PE-conjugated isotype controls were from BD (Franklin Lake, NJ). For quantitative polymerase chain reaction (qRT-PCR), we used RNeasy kit from QIAGEN (Redwood City, CA). All primers were purchased from Bio-Rad (Hercules, CA). Cell culture media, cell culture related products, and western blot reagents were obtained from Thermo Fischer Scientific (Waltham, MA).

### Semi high-throughput screening

We undertook a semi high-throughput screening for antagonists of Trpv4 from a library of 2000 natural compounds (MSSP-2000, Johns Hopkins Chemcore) using a fluorometric imaging plate reader (FLIPR)-based calcium influx assay^14,17^. We identified ginkgetin (GGT), as a novel antagonist to TRPV4. Primary and secondary screening was performed with RAW264.7, HDF, and BMDMs to assess the ability of ginkgetin and other candidates to inhibit TRPV4-elicited Ca^2+^ influx^14,17^. As controls, we used A23187, a calcium ionophore, TRPV4 null BMDMs, and GSK219, a selective TRPV4 antagonist^17^. After secondary screening, we identified six compounds showing more than threefold inhibition of TRPV4-mediated Ca^2+^ influx compared to vehicle control. Ginkgetin was identified as the most potent inhibitor of TRPV4 activity. BMDMs (5 × 10^4^ cells/well) were seeded into collagen coated (10 µg/ml) 96-well clear bottom black plates, and incubated at 37 °C, 5% CO_2_. On the day of the experiment, cells were loaded with calcium 6 dye in HBSS, 20 mM HEPES, 2.5 mM probenecid and incubated for 45 min at 37 °C. After incubation, library compounds were added to each well to a final concentration of 2 µM. Plates were incubated for another 45 min at 37 °C. Ca^2+^ influx was induced by the addition of 10 nM of TRPV4 agonist GSK1016790A in vehicle- or compound-pretreated cells, and recorded by measuring ΔF/F (Max–Min), as previously described^17^. Data are shown as relative fluorescence units (RFU).

### Immunoblots

Thioglycolate-elicited peritoneal macrophages were seeded in 10% FBS containing DMEM and incubated overnight. Unadhered cells were washed off, and 1% bovine serum albumin (BSA) containing DMEM was added to the plate and incubated for 3 h before ginkgetin treatment. To determine expression of CD36, TRPV4, TLR2, TLR4, TLR6, and GAPDH proteins whole cell lysates were prepared from cells treated overnight with ginkgetin (1 and 5 µM) or vehicle in the presence or absence of oxLDL (25 µg/ml). To determine the expression levels of LPS-triggered p-JNK and JNK, cells were pretreated with ginkgetin (5 and 10 µM) for 3 h and then stimulated with E. coli LPS for 30 min. Whole cell lysates were prepared from twice-washed cells (ice cold PBS) using RIPA buffer with added protease-inhibitor cocktail (Thermo Scientific, MA). Blots were probed with rabbit anti-TRPV4 (Alomone Lab, Jerusalem, Israel), anti-mouse CD36 (R&D, Minneapolis, MN), rabbit anti-TLR4, rabbit anti-TLR6, rabbit anti-JNK, and rabbit anti-p-JNK primary antibodies (Cell Signaling, Danvers, MA) followed by anti-rabbit and anti-mouse HRP-conjugated secondary antibodies (R&D, Minneapolis, MN). Blots were stripped and re-probed with anti-GAPDH IgG (1:2000; Santa Cruz, Dallas, TX) for loading control.

### Foam cell formation

Thioglycolate-triggered MRM were seeded on glass coverslips in a 12 well plate with DMEM, 10% FBS and incubated at 37 °C, 5% CO_2_ for 2 h. GGT (1 or 10 µM) was added to the cells, and after 3 h of incubation, oxLDL (50 µg/ml) was added, and cultures were incubated overnight at 37 °C. Native LDL (50 μg/ml) was added to control group wells and incubated overnight. Cells were fixed in 4% paraformaldehyde followed by Oil Red O staining to quantify foam cell generation.

### Adenovirus vector transduction

We collected Adenovirus constructs to express Ad(RGD)-mouse-TRPV4 (Ade-TRPV4; ~10^10^ pfu/ml), and adenovirus blank vector, Ad(RGD)-CMV-null (Ade-Vec; 10^11^ pfu/ml), from Vector Biolabs, Malvern, PA. We used 1 × 10^8^ pfu/ml for all experiments. For foam cell generation studies, mouse peritoneal macrophages were incubated with Ade-TRPV4 or Ade-Vec in DMEM media for 72 h before addition of oxLDL to generate macrophage foam cells.

### Binding and uptake of oxLDL

To asses binding, peritoneal macrophages were treated with two doses (1 or 10 µM) of ginkgetin or vehicle for 3 h and incubated with DiI-oxLDL at 4 °C for one hour. After pre-treatment with ginkgetin or vehicle, cells were incubated with DiI-oxLDL at 37 °C for 30 min to asses uptake. Images were captured by fluorescence microscopy (63x), and Image J was used to quantify results.

### Flow cytometry analysis

Thioglycolate-elicited peritoneal macrophages were cultured in DMEM, 10% FBS for 24 h. Unadhered cells were washed off, serum free medium was added, and cells were incubated for 2 h followed by addition of 5 µM GGT or vehicle, and incubation was continued for 3 h. Treated cells were scraped off the plate and resuspended in PBS, 2% BSA. Cells were incubated with primary antibodies for 45 min followed by 30 min incubation with PE-conjugated secondary antibody. A FACSCantoII flow cytometer (BD Bioscience, Ontario, Canada) was used to acquire data. All data were processed using FlowJo software.

### qRT-PCR

Thioglycolate-induced macrophages were treated with 5 µM ginkgetin or vehicle for 3 h; 25 µg/ml of oxLDL were added to vehicle or ginkgetin-treated cells, and incubation was continued overnight. RNeasy Micro Kit (#74,004) from QIAGEN was used to extract total RNA, and iTaq Universal SYBR Green One-Step Kit from Bio-Rad was used to reverse transcribe the RNA. A C1000 Touch Thermal Cycler (Bio-Rad) was used to acquire data. Expression of a gene was determined as the amount of gene-specific mRNA relative to GAPDH mRNA using the comparative C_T_ method described in the Bio-Rad qRT-PCR system user bulletin.

### Statistical analysis

Data are presented as mean ± SEM of biological triplicates. GraphPad Prism 7.0 software was used, and calculations were conducted using Student’s t-test for two groups or one-way ANOVA for multiple groups.

### Ethical approval

All experiments on mice were conducted in accordance with the Institutional Animal Care and Use Committee (IACUC) guidelines, and were approved by the University of Maryland‐College Park Review Committee.

## Results

### Semi high-throughput screening for antagonists of TRPV4 from a library of natural compounds using a FLIPR-based Ca^2+^ influx assay

**Figure 1:**
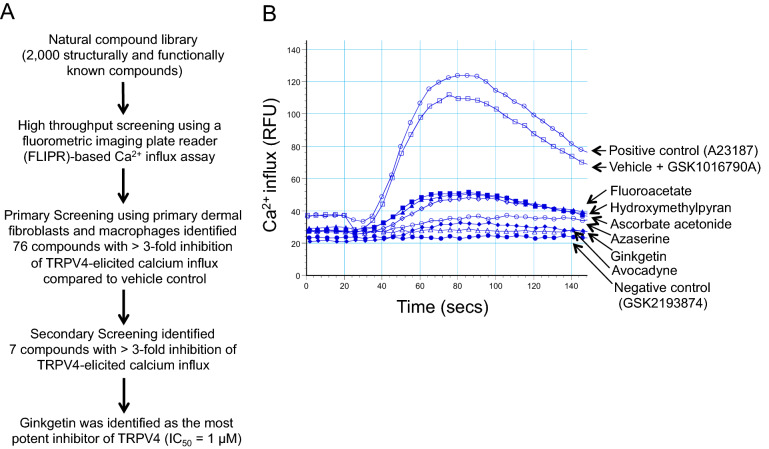
Semi high-throughput screening-based identification of ginkgetin as a novel TRPV4 inhibitor from a library of natural compounds. (**A**) Flow chart showing the workflow resulting in identification of ginkgetin as a potential TRPV4 inhibitor from a library of 2000 natural compounds. (**B**) Representative FlexStation 3 recording showing the inhibitory effect of 6 natural compounds on TRPV4 agonist (GSK1016790A)-induced Ca^2+^ influx in calcium 6 dye-loaded BMDMs during secondary screening. Pretreatment with all 6 compounds showed inhibitory effects on TRPV4-dependent Ca^2+^ influx compared to cells pretreated with vehicle (negative control; vehicle plus GSK1016790A (10 nM)) or calcium ionophore (A23187, 2 μM). We used selective TRPV4 antagonist, GSK2193874 (10 nM), as a negative control. All experiments were repeated three times in quadruplicate. RFU: relative fluorescence unit.

A library containing a collection of structurally and functionally known natural compounds was screened using a semi high-throughput FLIPR-based Ca^2+^ influx assay (Fig. 1A) to investigate the modulatory effect of these compounds on TRPV4-elicited Ca^2+^ influx. Initial screening performed with 2000 compounds on primary human dermal fibroblasts and RAW264.7 cells identified 76 lead compounds that significantly (≥ threefold) inhibited GSK101-induced (a TRPV4 specific agonist) Ca^2+^ influx. We performed a secondary screening of the lead compounds using the same assay on RAW 264.7 cells. We further verified and selected 6 compounds which produced reproducible and significant (> threefold; p < 0.001) inhibition of TRPV4-elicited Ca^2+^ influx in BMDMs as compared to the vehicle control (Fig. 1B). Our screening assay proved to be a high confidence assay for detecting small molecule compounds with TRPV4 inhibitory effects as reflected by a good Z factor value (0.703). We have used A23187 (A23), a calcium ionophore, as the positive control, and GSK2193874 (GSK219), a selective small molecule chemical inhibitor of TRPV4, as the negative control^14–17^. Ginkgetin (GGT) was identified as one of the most potent inhibitors of TRPV4 in the screening. Since previously published reports showed that GGT has an anti-inflammatory and anti-adipogenic effect, we selected GGT for further functional analysis on TRPV4-dependent macrophage proatherogenic activity^48,49,52,53^.

### TRPV4 inhibition by GGT abrogates oxLDL-induced macrophage foam cell formation

**Figure 2:**
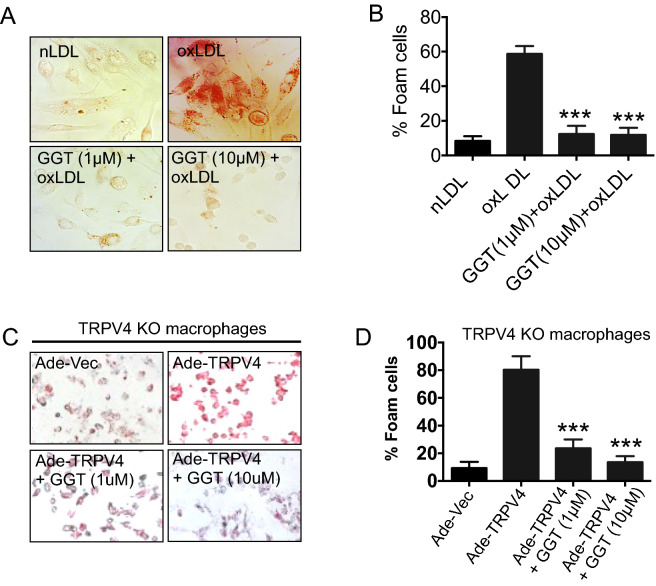
Inhibition of TRPV4 by ginkgetin abrogates oxLDL-induced macrophage foam cell formation. (**A**) Thioglycolate-induced murine peritoneal macrophages were treated overnight with 50 µg/ml oxLDL, followed by a 3 h preincubation with vehicle or 1 or 10 µM ginkgetin. Cells were treated with 50 µg/mL native LDL (nLDL) as control. Original magnification: 40x. (**B**) Quantification of the results shown in (**A**). n = 500 cells/condition. Student’s *t*-test; ***p < 0.001. Data represent ± SEM. (**C**) TRPV4 KO RPMs were transduced with either Ade-Vec or Ade-TRPV4, and were untreated or treated with GGT in the presence of oxLDL for 16 h on day 5 of transduction for macrophage foam cell formation. (**D**) Quantification of the results shown in C. n = 100 cells/condition. Student’s *t*-test; ***p < 0.001.

Our previous studies showed that TRPV4-elicited Ca^2+^ influx is involved in oxLDL-mediated foam cell formation ^14,16^. Therefore, we evaluated the possibility that foam cell formation was inhibited by GGT-mediated antagonization of TRPV4 activity. Wild-type murine peritoneal macrophages were incubated with oxLDL to induce foam cell formation in the presence or absence of GGT, and analyzed by Oil Red O staining. As expected, we found that generation of foam cells increased by fivefold in oxLDL treated macrophages in comparison to native LDL (Fig. 2A,B). However, treatment of the macrophages with GGT (1 or 10 µM) prior to stimulation with oxLDL significantly decreased the percentage of foam cells (Fig. 2A,B). Collectively, these findings suggest an inhibitory effect of GGT on macrophage foam cell formation possibly targeting TRPV4-elicited Ca^2+^ influx. Furthermore, we overexpressed TRPV4 in TRPV4 null macrophages by using adeno-virus construct and then induced foam cell formation by using oxLDL in the presence of GGT. We found that overexpression of TRPV4 increased the foam cell formation which was blocked when we pretreated the cell with GGT, suggesting that inhibition of the proatherogenic foam cell formation by GGT is TRPV4 dependent (Fig. 2C,D).

### GGT does not block basal level of expression of TRPV4 and inflammatory receptors

**Figure 3:**
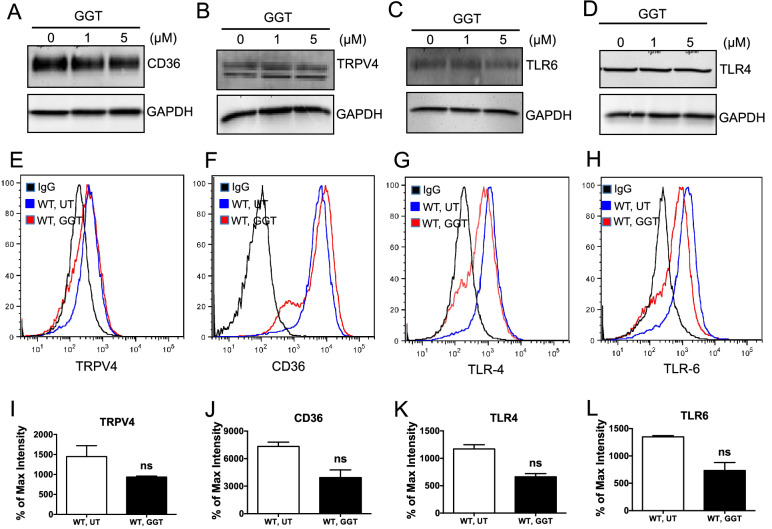
Ginkgetin treatment does not alter total protein or surface expression of TRPV4, CD36, and TLRs in macrophages. (**A**–**D**) Immunoblots of macrophage whole cell lysates following overnight treatment with 1 or 5 µM ginkgetin or vehicle. Representative immunoblots show total protein expression of CD36 (**A**), TRPV4 (**B**), TLR6 (**C**) and TLR4 (**D**) in ginkgetin-treated and untreated cells. Three independent biological replicates and 3 experimental repeats for each set of experiment were performed. (**E**–**H**) Flow cytometric analysis of macrophages treated overnight with 5 µM ginkgetin or untreated showing surface expression of TRPV4 (**E**), CD36 (**F**), TLR4 (**G**), and TLR6 (**G**) in untreated and ginkgetin-treated cells. Three independent biological replicates and 30,000 cells per condition were analyzed. (**I**–**L**) Quantitative analysis of results from (**E**–**H**). Analyzed by Two-tailed t-test with 99% confidence interval. Cells counted per experiment, 30,000; two experimental repeats.

Scavenger receptor CD36 plays a major role in the uptake and binding of oxLDL, and foam cell formation, critical processes in atherosclerosis^4–7,54,55^. Recently, a growing body of evidence suggests the involvement of toll-like receptors in atherosclerosis^2,4,56^. To investigate whether GGT blocked foam cell formation via affecting the expression of CD36, TLR4, TLR6, and TRPV4, we performed immunoblot analysis at two concentrations of GGT (1 or 5 µM). We found similar expression levels of CD36, TRPV4, TLR6, and TLR4 as compared to the untreated control (Fig. 3A–D). Flow cytometric analysis also revealed no significant difference in the cell surface expression of the proteins with or without GGT treatment (Fig. 3E–H, I–L). Considered all together, these results suggest that GGT blocks macrophage foam cell formation without inhibiting the basal levels of surface expression of TRPV4, CD36, TLR4, and TLR6.

### GGT blocks oxLDL-induced expression of TRPV4 in macrophages

**Figure 4:**
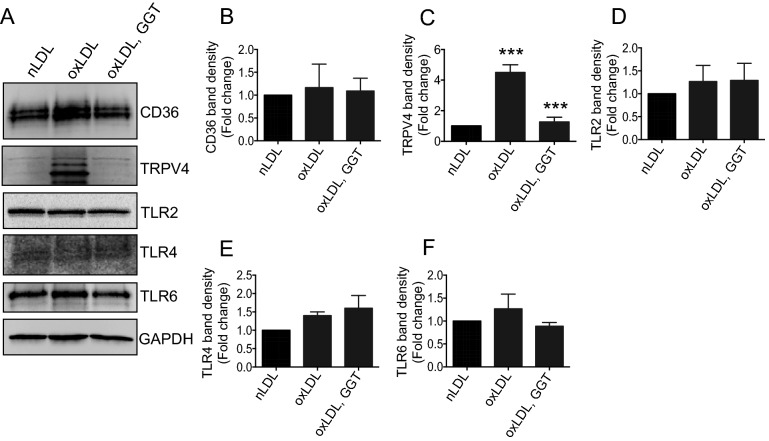
Ginkgetin blocks oxLDL-induced upregulation of TRPV4 proteins in macrophages. Whole cell lysates were prepared from macrophages pretreated with vehicle or 10 µM ginkgetin for 3 h followed by overnight incubation with 50 µg/mL of oxLDL or nLDL. Representative immunoblots (**A**) show total protein expression of CD36, TRPV4, TLR2, TLR4, TLR6, and GAPDH in ginkgetin-treated and untreated cells in the presence of oxLDL or nLDL. Quantitative analysis of CD36 (**B**), TRPV4 (**C**), TLR2 (**D**), TLR4 (**E**), and TLR6 (**F**) proteins shown in (**A**). Student’s *t*-test, ***p < 0.001, Data represent ± SEM.

As our previously published studies showed that TRPV4-elicited Ca^2+^ plays a role in oxLDL-mediated macrophage foam cell formation^14,16^, we sought to determine if GGT blocked foam cell formation via suppression of oxLDL-induced expression of CD36, TLR2, TLR4, TLR6 or TRPV4. We found that overnight stimulation of macrophages with oxLDL resulted in significant upregulation of the expression of TRPV4 proteins compared to nLDL, while expression of other receptors (CD36, TLR2, TLR4, and TLR6) remained unchanged (Figs. 4A–F). Pretreatment of cells with GGT prior to stimulation with oxLDL selectively decreased the level of expression of TRPV4 proteins compared to vehicle treatment (Fig. 4A–F). Collectively, these findings suggest an inhibitory effect of GGT on TRPV4 protein expression, which may be in part involved in suppression of foam cell formation by GGT.

### Uptake of oxLDL, but not binding by macrophages is suppressed by GGT

**Figure 5:**
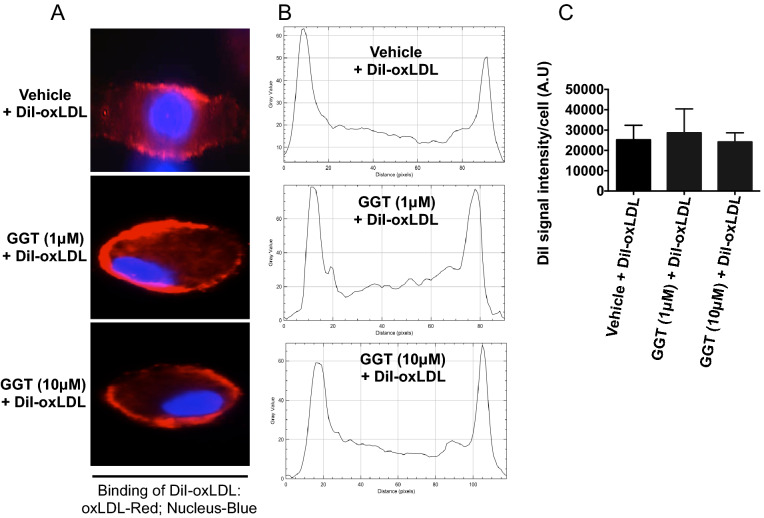
Ginkgetin does not suppress DiI-oxLDL binding to macrophages. Macrophages were pretreated with vehicle or ginkgetin (1 or 10 µM) for 3 h followed by incubation with DiI-labeled oxLDL (2.5 µg/mL) for 1 h at 4 °C. (**A**) Representative fluorescence microscopic images (original magnification, 63×) of DiI-oxLDL binding on macrophage membrane surface (n = 20 cells/condition). (**B**) Results from experiment A shown as plot profile using NIH ImageJ software. (**C**) Quantitative analysis of results from A. n = 20 cells/condition.

**Figure 6:**
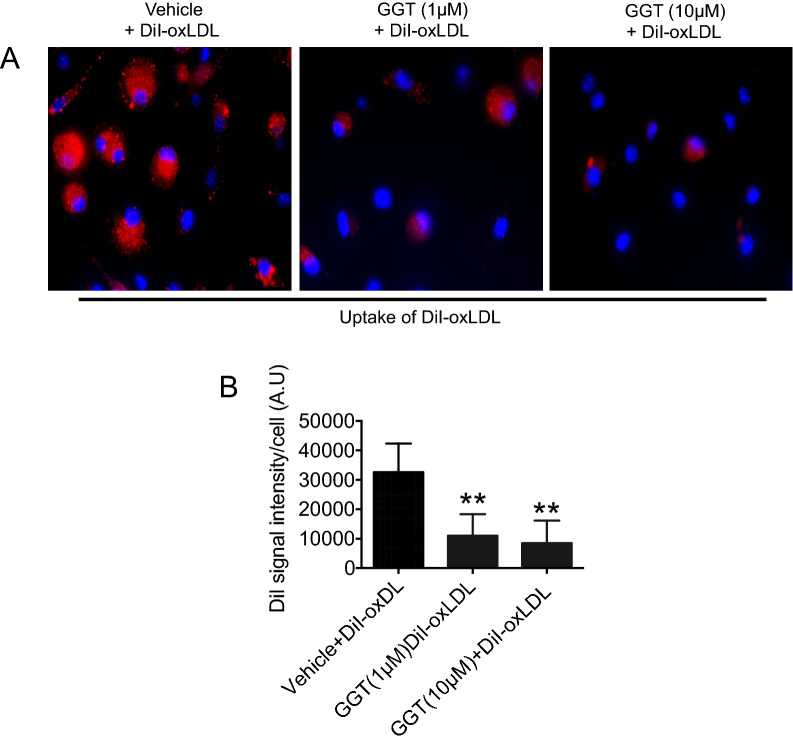
Ginkgetin blocks DiI-oxLDL uptake in macrophages. Macrophages were pretreated with vehicle or two doses of ginkgetin (1 or 10 µM) for 3 h followed by incubation with DiI-labeled oxLDL (2.5 µg/mL) for 30 min at 37 °C. (**A**) Representative images of DiI-oxLDL from ten different fields per condition (original magnification, 40×) are shown. DiI-oxLDL uptake indicated by red fluorescence. (**B**) Bar graphs show mean DiI fluorescence intensity (mean ± SEM) analyzed using NIH ImageJ software. **p < 0.01 for oxLDL uptake in untreated versus ginkgetin treated cells; n = 50 cells/condition.

Because TRPV4 has been previously shown to have a role in uptake of oxLDL and foam cell formation^14,16^, we evaluated whether treatment with GGT caused impairment in the binding of oxLDL on the macrophage surface. WT peritoneal macrophages were treated with GGT prior to incubation with DiI-oxLDL at 4 °C, and binding was assessed. The results indicate that binding of oxLDL on the macrophage surface was not significantly impeded by GGT (1 or 10 µM) treatment compared to vehicle control (Fig. 5A–C). Having established that GGT does not inhibit the binding of oxLDL, we next aimed to determine if uptake of oxLDL in macrophages was impaired by GGT treatment. Interestingly, our results indicated that there was significant inhibition in the uptake of oxLDL in macrophages in GGT pretreated cells as compared to the vehicle treated group (Fig. 6A,B). Considered all together, these results suggest that GGT blocks the uptake of oxLDL, but not binding, in macrophages.

### GGT blocks proatherogenic JNK2 activation and inflammation in macrophages

**Figure 7:**
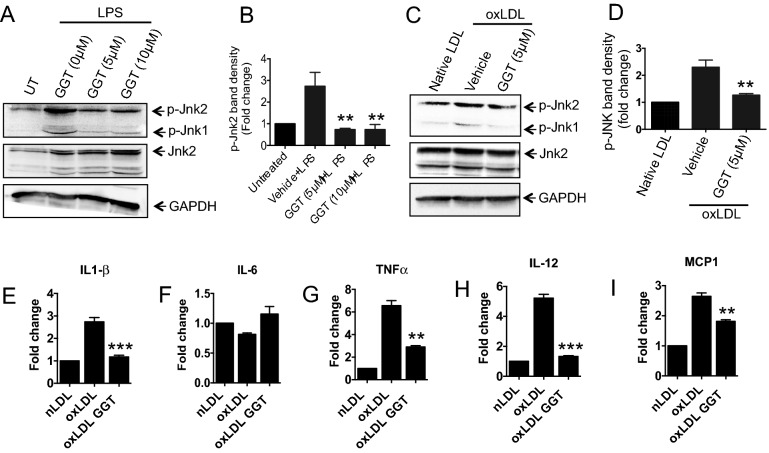
Ginkgetin blocks proatherogenic JNK2 activation and oxLDL-induced upregulation of proinflammatory gene expression in macrophages. Macrophages were treated with two doses of ginkgetin (5 or 10 µM) for 3 h followed by stimulation with *E. coli* LPS for 30 min. Immunoblot analysis was performed on whole cell lysates to evaluate phosphorylated JNK (p-JNK). (**A**) Representative immunoblot images showing levels of p-JNK1/2, total JNK, and GAPDH proteins (loading control). (**B**) Quantitative analysis of p-JNK2 levels after normalizing with total JNK-2 protein expression. Student’s *t*-test; **P < 0.01, mean ± SEM. (**C**) Representative immunoblot images showing levels of p-JNK1/2, total JNK, and GAPDH proteins in macrophages stimulated by oxLDL or native LDL. (**D**) Quantitative analysis of results from C. Student’s *t*-test; **P < 0.01, mean ± SEM. Two independent biological replicates and three experimental repeats for each set of data were performed. For qRT-PCR analysis, macrophages were treated with 10 µM ginkgetin for 3 h followed by oxLDL (50 µg/mL) overnight treatment at 37 °C. mRNA was harvested from the cells, and qRT-PCR was carried out using SYBR green gene expression assay to evaluate *Il1β* (**E**), *Il6* (**F**), *Tnfα* (**G**), *Il12* (**H**) and *Mcp-1* (**I**). n = 2 biological repeats and 3 experimental repeats. One-way ANOVA followed by Bonferroni’s multiple comparison test; **p < 0.01, ***p < 0.001.

Published reports from our laboratory and others have shown that activation of JNK2 plays a critical role in foam cell formation and atherosclerosis^7,57^. To determine if GGT could inhibit the activation of JNK1/2, macrophages were treated with GGT followed by stimulation with LPS or oxLDL. Results from immunoblot analysis showed that treatment with GGT significantly suppressed oxLDL- or LPS-induced phosphorylation of JNK1/2 as compared to the untreated or native LDL-treated control (Fig. 7A–D). It has been shown that JNK activation in macrophages induce transcription of proinflammatory mediators associated with atherosclerosis^58–60^. To determine if GGT could potentially block the expression of these proinflammatory regulators in macrophages, GGT-pretreated cells were stimulated with oxLDL, and mRNA expression levels of TNFα, IL 6, IL12, IL1β, and MCP1 were quantified by qRT-PCR analysis. We detected significant downregulation in oxLDL-induced expression levels of TNFα, IL12, IL1β, and MCP1 mRNA in GGT-treated cells compared to vehicle treated controls (Fig. 7E–I). Taken together, these results suggest that GGT blocks proatherogenic JNK2 activation and inflammatory gene expression in response to oxLDL stimulation in macrophages.

## Discussion

Natural compounds such as flavonoids and polyphenols are known to modulate cardiovascular responses via diverse mechanisms, such as inhibition of proinflammatory signals, insulin sensitization, oxidative status, and improving blood lipid profiles^40–45^. Herein, we report the semi high-throughput screening-based identification and functional characterization of ginkgetin, a biflavone, as a novel inhibitor of TRPV4-dependent proatherogenic/inflammatory processes in macrophages. We specifically showed that i) ginkgetin blocks TRPV4-mediated Ca^2+^ influx into macrophages, ii) ginkgetin blockade of TRPV4 function markedly decreased oxLDL-induced foam cell formation by suppressing the uptake of oxLDL in macrophages, and iii) ginkgetin blocks LPS- and oxLDL-induced phosphorylation of JNK1/2, expression of TRPV4 proteins, and inflammatory gene expression. In aggregate, the results of our study showed that ginkgetin inhibits proatherogenic/inflammatory macrophage function in a TRPV4-dependent manner.

Atherosclerosis, an aortic disease, is initially fueled by inflammation and the engorgement of macrophages with oxLDL, inducing formation of macrophage foam cells, which subsequently progress to form atheroma^2–10^. Since foam cell formation is a critical process in atherogenesis, understanding and manipulating foam cell formation can have therapeutic potential. Macrophages are known to express an array of membrane penetrating ion channels and pumps, including TRPV4, a mechanosensitive polymodal Ca^2+^ permeable ion channel, which is activated by both physical and biochemical stimuli^11–24^. Published studies by our laboratory and others have identified a role of TRPV4 in macrophage inflammation and oxLDL-induced foam cell formation^14–16,26^. TRPV4 can thus serve as a viable target for attenuation of proatherogenic processes.

Ample evidence suggests that natural compounds affect metabolic and inflammatory disorders like cardiovascular diseases by targeting fat and cholesterol synthesis, inhibiting prostaglandin synthesis, neutralizing reactive oxygen and nitrogen species, and impairing NF-κB-mediated release of proinflammatory cytokines^42–45,61^. However, the roles of natural compounds such as TRPV4 antagonists have not been investigated until recently. A study by Wang et al*.* reported that berberine, an alkaloid from Chinese herbs, mediates vasorelaxation by suppression of TRPV4 function^46^. Other groups have reported that plant cannabinoids can regulate the expression and activity of TRPV4 channels, and can have potential therapeutic applications in the gastrointestinal tract and other TRPV4-associated channelopathies^62^. Ma et al. recently proposed that apigenin, a plant-derived flavone, activates TRPV4 resulting in vascular dilation^47^. Herein, we report that ginkgetin, a biflavone, identified using semi high-throughput screening, blocks oxLDL-induced foam cell formation and inflammatory signals by targeting a TRPV4-evoked proatherogenic/inflammatory pathway. Ginkgetin, isolated from Gingko biloba, is a known anti-inflammatory and anti-carcinogenic compound^48,49^. Studies by Lian et al*.* have highlighted a role of ginkgetin in improving atherosclerosis by reducing MMP-2 and MMP-9 and increasing levels of NO and NOS in thoracic aortas of rat^52^. In the present study, we showed that ginkgetin blocks TRPV4-elicited Ca^2+^ influx in macrophages. Mechanistically, we showed that ginkgetin blocks the uptake of oxLDL, but not binding, in macrophages.

Studies done in vivo and in vitro suggested a critical role of CD36 as the principal scavenger receptor in uptake of oxLDL and macrophage foam cell formation^2–7^. It was shown that CD36 null mice that lack ApoE or LDLR are less prone to developing atherosclerotic lesions^5,6^. Previous studies from our group identified an essential role of the CD36-JNK signaling cascade in macrophage foam cell formation^7^. Toll like receptors TLR2, TLR4, and TLR6 act as coreceptors by interacting with CD36, and have been shown to be involved in atherogenesis^2,56,63^. We examined the possibility that lack of foam cell formation seen in ginkgetin-treated macrophages was due to decreased expression of CD36, TRPV4, TLR2, TLR4, or TLR6 proteins. Interestingly, ginkgetin treatment did not significantly decrease expression of CD36, TLR2, TLR4, or TLR6 proteins at basal levels or under oxLDL-stimulated conditions. However, ginkgetin specifically blocked oxLDL-induced upregulation of expression of TRPV4 proteins, while its expression at basal levels remained unchanged. Considered all together, these results suggest that ginkgetin blocks oxLDL-induced foam cell formation via a mechanism independent of CD36 and TLR expression, but dependent on TRPV4.

Previous studies from our group and others showed that JNK2 activation is involved in foam cell formation and atherosclerotic lesion development^7,57^. It was also reported that JNK is involved in the modulation of MMP-9 and MMP-13, which are overexpressed in atherosclerotic lesions^64–68^. Given the recognized link of JNK in atherogenic processes, we wanted to determine if ginkgetin could inhibit the activation of JNK. In accord with previous reports, our results also showed that ginkgetin blocks inflammatory stimulus-induced phosphorylation of JNK2 in macrophages. This result suggests a possible mechanism by which ginkgetin blocks foam cell formation. Furthermore, we found a significant downregulation in oxLDL-induced expression of TNFα, IL12, IL1β, and MCP1 mRNA in ginkgetin-treated cells compared to vehicle control, highlighting potential anti-inflammatory roles of ginkgetin in response to oxLDL. In summary, our results showed that ginkgetin inhibits proatherogenic and inflammatory macrophage function in a TRPV4-dependent manner. These findings thus strengthen the rationale for the use of natural compounds in development of potential therapeutics and/or chemopreventive reagents.

## Supplementary Information

Supplementary Information
