# Supplementary material for: Identification and functional analysis of a biflavone as a novel inhibitor of transient receptor potential vanilloid 4-dependent atherogenic processes
Source: Sci Rep. 2021 Apr 14;11:8173. doi: 10.1038/s41598-021-87696-9 (PMC8047007; doi:10.1038/s41598-021-87696-9)
Supplement: Supplementary file 1 — Supplementary Information [file 41598_2021_87696_MOESM1_ESM.pdf]

Identification and functional analysis of a biflavone as a novel inhibitor of Transient Receptor Potential Vanilloid 4-dependent atherogenic processes

Mazen O. Alharbi[1], Bidisha Dutta[1], Rishov Goswami[1], Shweta Sharma[1], Kai Y. Lei[1], Shaik O. Rahaman[1]

[1]University of Maryland, Department of Nutrition and Food Science, College Park, MD 20742

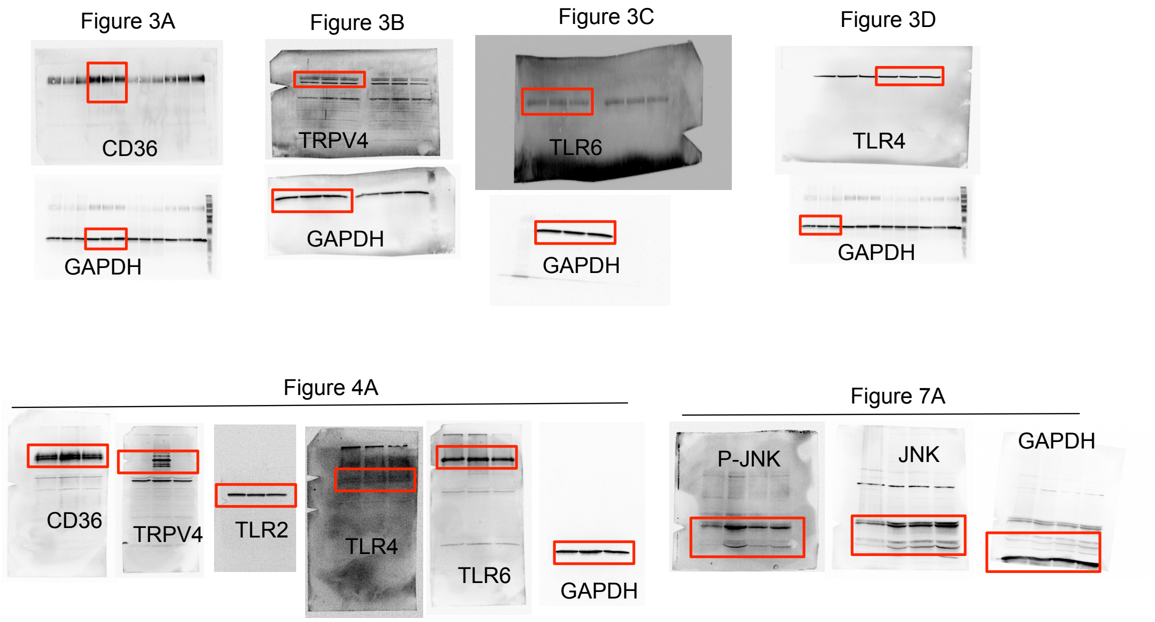

Supplementary Figure 1. **Immunoblots of the cropped image shown in Figure 3A-D, Figure 4A, and Figure 7A.** The cropped areas on the blots are indicated with red-color boxes.
